# Development of a novel strategy for robust synthetic bacterial promoters based on a stepwise evolution targeting the spacer region of the core promoter in *Bacillus subtilis*

**DOI:** 10.1186/s12934-019-1148-3

**Published:** 2019-05-29

**Authors:** Laichuang Han, Wenjing Cui, Feiya Suo, Shengnan Miao, Wenliang Hao, Qiaoqing Chen, Junling Guo, Zhongmei Liu, Li Zhou, Zhemin Zhou

**Affiliations:** School of Biotechnology, Key Laboratory of Industrial Biotechnology (Ministry of Education), Jiangnan University, Wuxi, 214122 Jiangsu China

**Keywords:** Promoter engineering, *Bacillus subtilis*, Synthetic biology, Stepwise evolution

## Abstract

**Background:**

Promoter evolution by synthetic promoter library (SPL) is a powerful approach to development of functional synthetic promoters to synthetic biology. However, it requires much tedious and time-consuming screenings because of the plethora of different variants in SPL. Actually, a large proportion of mutants in the SPL are significantly lower in strength, which contributes only to fabrication of a promoter library with a continuum of strength. Thus, to effectively obtain the evolved synthetic promoter exhibiting higher strength, it is essential to develop novel strategies to construct mutant library targeting the pivotal region rather than the arbitrary region of the template promoter. In this study, a strategy termed stepwise evolution targeting the spacer of core promoter (SETarSCoP) was established in *Bacillus subtilis* to effectively evolve the strength of bacterial promoter.

**Results:**

The native promoter, P_srfA_, from *B. subtilis*, which exhibits higher strength than the strong promoter P43, was set as the parental template. According to the comparison of conservation of the spacer sequences between − 35 box and − 10 box among a set of strong and weak native promoter, it revealed that 7-bp sequence immediately upstream of the − 10 box featured in the regulation of promoter strength. Based on the conservative feature, two rounds of consecutive evolution were performed targeting the hot region of P_srfA_. In the first round, a primary promoter mutation library (pPML) was constructed by mutagenesis targeting the 3-bp sequence immediately upstream of the − 10 box of the P_srfA_. Subsequently, four evolved mutants from pPML were selected to construction of four secondary promoter mutation libraries (sPMLs) based on mutagenesis of the 4-bp sequence upstream of the first-round target. After the consecutive two-step evolution, the mutant P_BH4_ was identified and verified to be a highly evolved synthetic promoter. The strength of P_BH4_ was higher than P_srfA_ by approximately 3 times. Moreover, P_BH4_ also exhibited broad suitability for different cargo proteins, such as β-glucuronidase and nattokinase. The proof-of-principle test showed that SETarSCoP successfully evolved both constitutive and inducible promoters.

**Conclusion:**

Comparing with the commonly used SPL strategy, SETarSCoP facilitates the evolution process to obtain strength-evolved synthetic bacterial promoter through fabrication and screening of small-scale mutation libraries. This strategy will be a promising method to evolve diverse bacterial promoters to expand the toolbox for synthetic biology.

**Electronic supplementary material:**

The online version of this article (10.1186/s12934-019-1148-3) contains supplementary material, which is available to authorized users.

## Background

*Bacillus subtilis* is a nonpathogenic bacterium that is free of exotoxins and endotoxins and can be used for the over-production of several kinds of heterologous proteins as a generally recognized as safe (GRAS) microorganism [1]. Over the past decades, *B. subtilis* has comprehensively been developed as a workhorse for the secretory over-production of numerous recombinant proteins and high value-added chemicals that are widely used in industrial biotechnology [1–4]. Several industrially and pharmaceutically used recombinant proteins are biosynthesized and actively exported into the extracellular milieu, employing powerful genetic elements and native highly efficient secretory translocation systems [5–10]. It also naturally produces many valuable biochemicals, such as poly-γ-glutamic acid, riboflavin, surfactants and antimicrobial peptides [11–14]. After successfully sequencing of the genome of *B. subtilis* 168 [15], more and more groundbreaking studies focusing on the fundamental sciences, such as biosynthetic pathways, the omics-driven system biology for metabolism [16–18], the cell–cell signaling pathway [19], and the mechanisms of controlling key metabolic intersections [20], have been performed. More importantly, booming technologies in *B. subtilis*, such as the DNA assembly [21], the CRISPR/Cas9 and ssDNA strategies for genome editing [22–24], and large-scale genome reduction [25], etc., provide a great push for developing *B. subtilis* into an ideal chassis for synthetic biology.

In synthetic biology, it is generally regarded that the designer functions in living chassis are performed by gene circuits, which directly exert diverse tailor-made devices, such as tunable oscillations [26], Boolean logic [27, 28] and pattern formation [29] and so on. These achievements rely on gene circuits of increasing size and complexity so that biological engineers must finely adjust the expression level of many different genes at a time. Although robust synthetic circuits are required to build well-characterized biological parts, the incomplete characterization of the promoters, the repressors, the ribosome binding sites, and the terminators in the chassis renders uncertainties, resulting from an unstable performance in a distinct genetic context [30]. These versatile concerns impede the systematical development of *B. subtilis* into a robust synthetic chassis. Therefore, reliable and stable biological parts are the essential prerequisite to fully exploiting the capability of the bacteria in synthetic biology.

Recently, diverse biological parts, comprising synthetic promoters, Ribosome Binding Sites (RBS), protein degradation tags SsrA [31], and small RNA-based regulators and switches [7, 32, 33], were constructed and directedly engineered to precisely tune the gene expression in *B. subtilis*. Among these synthetic biological parts, the promoter is the primary genetic pivotal element for gene expression, since it controls gene expression at the most fundamental level and determines the spatiotemporal regulation. Given the extremely important role of the promoter in the design genetic circuits in synthetic biology, largely broadening the promoter toolbox in *B. subtilis* enables the effective design of more complex circuits to perform diverse customized behaviors, such as those in *E. coli*. Generally, the strong constitutive promoters, namely, P43 and P_veg_, have been widely used in these simple systems to produce bulky industrial enzymes [34–36]; however, some typical inducible promoters, such as P_xylA_ and P_spac_, are sometimes employed according to the specific instances [37, 38]. Theoretically, an inducible promoter achieves the desired levels of gene expression by modulating the inducer concentration. However, in several instances, especially when more complex circuits that require two or more inducers are constructed, uncertainties and variations arise, due to leaky expression before induction and non-degradable chemicals after induction [30]. A reliable and stable constitutive promoter is of great importance as an alternative biological part that is involved in differentially regulating networks of complex circuits, such as the regulation and rewiring of modular metabolic pathways in *B. subtilis* [39, 40].

Even though strong constitutive promoters have been well-characterized and utilized in several customized synthetic systems, there are also numerous drawbacks limiting their broad application in synthetic biology. Natural promoter activity is often context-specific and subject to interaction with a multitude of regulatory proteins, rendering the prediction of activity levels under varying conditions [41]. Since it is of great importance to predict and tune the activity and manner of the promoter in the host, the development and fabrication of orthogonal and robust promoters with a predictable expression manner are paramount to synthetic biology [41]. The basic structure of a prokaryotic promoter includes the UP element, the − 35 and − 10 boxes and the transcription start site. Among these regions, the UP element and the − 35/− 10 box mainly influence promoter activity [42–44]. Promoters are recognized by different sigma factors, based on the different consensus of the − 35/− 10 box [45, 46]. There are large quantities of frameworks that have been developed to produce the synthetic promoter library [41, 47]. The typical strategies, among these frameworks, are directed evolution and semi-rational design. Directed evolution, targeting the flaking region surrounding the consensus motif, is performed by degenerating the spacer between the − 35 box and the − 10 box, producing a promoter library with a large mutant capacity [48–50]. This strategy requires a high-resolution screening method to identify sufficiently large transformants to ensure that the desired variants are obtained [51]. It is a tedious and time-consuming process. Moreover, it requires an iterative identification to authenticate the real activity in the different genetic context of the host. Another simplified strategy is to construct hybrid and tandem promoters, which are categorized into the semi-rational design. In this strategy, the activity and sequence features of the parental promoter are usually well characterized so that the core region can be genetically fused in several repeats [6, 52]. This strategy is more convenient, since it does not require the construction and screening of a large library. However, although these strategies for promoter engineering modulate the transcription level, through the variant with the desired activity, the effective variants only output discrete transcriptional activities, which is unable to achieve the fine tuning of gene expression in complex gene circuits in some rigorous instances.

Thus, in this study, we developed a novel pipeline, which was termed the Stepwise Evolution Targeting Spacer region of Core Promoter (SETarSCoP) strategy using P_srfA_ as parental promoter, to efficiently evolve synthetic promoters in *B. subtilis*. The pipeline initiated with the construction of a stepwise semi-rational directed evolution. Accordingly, we obtained a series of mutant promoters with high activity and applied the strongest promoter to over express β-glucuronidase (GusA) from *E. coli* and nattokinase (NK) from *Bacillus natto*. In addition, we also successfully evolved the strength of both the constitutive promoter P_ylbP_ and the xylose-inducible promoter P_xylA_ using this strategy, manifesting that SETarSCoP is a convenient and highly efficient method to evolve the bacterial promoters in synthetic biology.

## Results and discussion

### Construction and quantification of promoter mutation libraries (PMLs) by two-round evolution via SETarSCoP

In this study, we constructed a semi-rationally directed evolution method, termed stepwise evolution targeting the spacer of the core region of promoter (SETarSCoP), to quickly and conveniently fabricate a robust promoter derived from a known parental template. This strategy was designed by the selective randomization of the “spacer” sequence between the − 35 box and the − 10 box where transcription level can be modulated by diversified nucleotide sequences. Although promoter engineering toward the spacer region has been reported previously in several cases, versatile strategies for those mutagenesis were generally targeting the entire spacer, typically between the − 35 box and − 10 box and the UP element [48, 53, 54]. The generated synthetic promoter library (SPL), containing a huge number of variants, is time consuming and laborious to screen. Specifically, it requires the state-of-the-art equipment, fluorescent activated cell sorting (FACS). In the process of the development of robust and synthetic promoters in *B. subtilis* in our previous studies [6, 55–57], a critical issue concerning the effective pipeline for the fabrication of highly efficient promoters spurred us to exploit distinct reliable pipelines that are more convenient to perform than the ordinary strategies. Importantly, previous studies indicate that the optimization of the − 16 region, which is located upstream of the − 10 box, substantially influences the transcription level of a bacterial promoter [58]. Our recent study on the semi-rational engineering of the promoter P_srfA_ from native *B. subtilis* further manifested that the diversification of the − 16 region significantly altered the activity of this promoter [56].

**Fig. 1:**
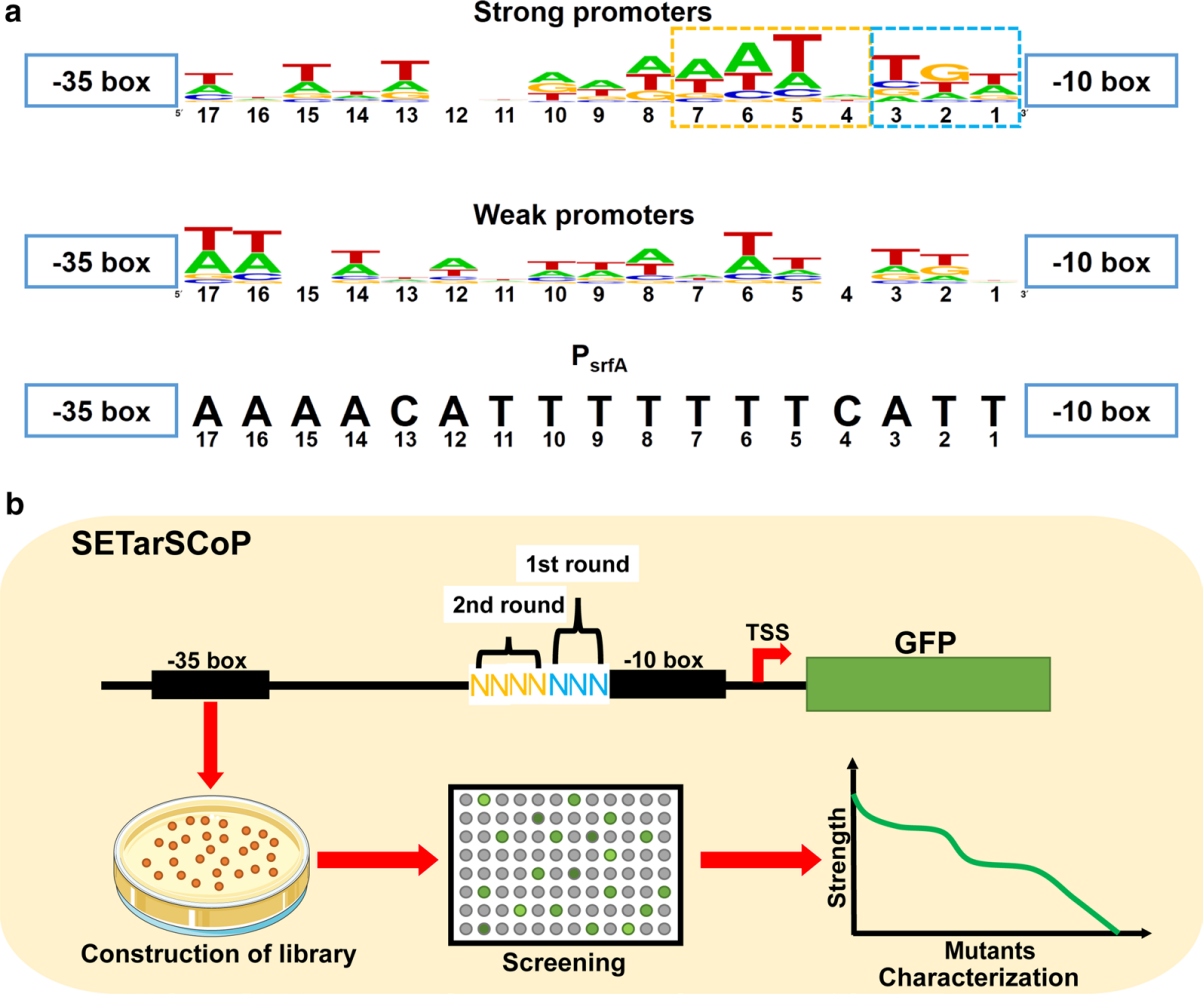
Schematic diagram of the SETarSCoP strategy. **a** Conservation analysis of spacer sequences between − 35 box and − 10 box of strong promoters and weak promoters. Conserved sequence was generated by WebLogo (http://weblogo.berkeley.edu/logo.cgi). The target regions for mutagenesis in the first and the second round of evolution were encircled by blue and orange dashed rectangle, respectively. **b** The scheme of SETarSCoP strategy performed by two rounds of evolution. The 3-bp sequence upstream of the − 10 box was randomly mutated at the first-round evolution. After screening, the positively evolved (being stronger than the parental promoter) mutants were chosen and validated. Subsequently, these evolved mutants were used as templates, 4-bp sequence immediately upstream of the region of the first-round mutation was used to perform random mutagenesis in the second-round evolution

Here, we constructed two promoter sets, which were composed of the 50 strongest and the 50 weakest σ^A^-dependent promoters from *B. subtilis* 168, respectively, to analyze the transcriptional levels by heatmap based on the data reported by Evert-Jan et al. [59] (Additional file 1: Figure S1). By aligning their spacer sequences between − 35 box and − 10 box, we found that the 7-bp region immediately upstream of the − 10 box displayed prominent conservation in the strong promoter set compared with that of the weak promoter set. More importantly, this region can also be divided into two parts according to the consensus feature. One portion is the 3-bp at the − 16 box (immediately upstream of the − 10 box), which covered an featured “TGn” motif. Another portion is the 4-bp adjacent to the − 16 box, which was also conserved in the strong promoter set compared to that of the weak promoter set (Fig. 1a). These results indicate that the spacer sequence stretching over the 7-bp sequence is a substantial target responsible for the strength variation, termed the hotspots. The region covers a few but considerably important sequence. Thus, it is reasonable to infer that evolution of the *B. subtilis* promoter could be achieved through small-scale promoter mutation library (PML) targeting this region. If it is feasible, this strategy will enable the screening more efficient than screening the large SPL constructing by random mutagenesis of the ~ 17-bp sequence between − 10 box and − 35 box. Therefore, how to manipulate these hotspots to augment the strength based on the parental promoter is a critical issue to fabrication of synthetic promoter.

**Fig. 2:**
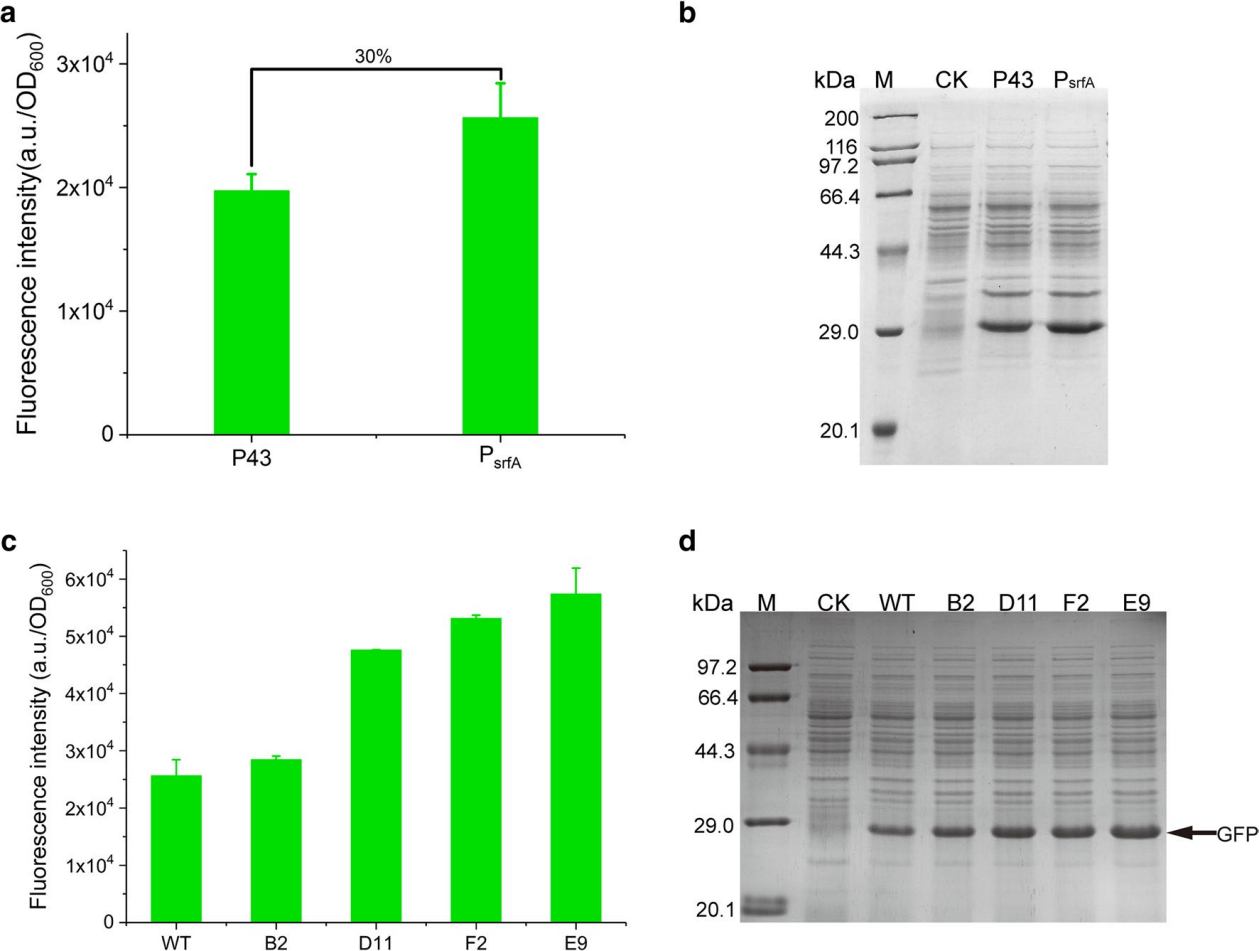
The mutagenesis and characterization of pPML. **a** Comparison of GFP expression driven by promoter P43 and P_srfA_. **b** SDS-PAGE analysis of GFP expression driven by P43 and P_srfA_. **c** Verification of the fluorescence intensity of the four representative mutants at the 24-h cultivation in the scale of the shake flask. The experiments were repeated independently in triplicate. The data were shown by mean ± S.D. **d** SDS-PAGE analysis of the GFP expression levels in the four selected mutants. The whole-cell proteins of each sample were separated by 12% SDS-PAGE and were then stained with Coomassie Brilliant Blue R-250. CK denoted the strain without any plasmid

To this end, we firstly sought to select the right parental template for evolution via SETarSCoP. At this stage, we compared the GFP expression driven by promoter P43 and P_srfA_, showing that the strength of P_srfA_ was about 1.3 times higher than that of P43 (Fig. 2a, b), indicating that the P_srfA_ is superior to P43. Therefore, we employed P_srfA_ as the parental promoter. Accordingly, we performed two-round random mutagenesis, sequentially targeting the 3-bp (ATT) at − 16 box and 4-bp (TTTC) adjacent sequence (Fig. 1b), generating two iterative small PMLs. GFP was employed to be the reporter protein to measure the promoter strength. We also constructed a genetically modified strain (*B. subtilis* comK) and optimized the component cell preparation to facilitate mutant library construction in *B. subtilis* (Additional file 1: Figure S2). The pool harboring the entire mutants was then transformed into *B. subtilis* comK, generating a primary promoter mutation library (pPML). We selected a total of 185 clones from the pPML, and determined the mutant strength by relative FI (a.u./OD_600_) at the mid-exponential growth phase. Overall, the pPML output a dynamic range of expression levels (Additional file 1: Figure S3A). Interestingly, amongst those mutants, 59 mutants had higher expression levels than the parental promoter, accounting for approximately one-third of the total mutants. Besides, five mutants among them had particularly higher expression levels than the parental promoter generally by more than 2 times (Additional file 1: Figure S3C). These data prominently indicate that evolution targeting the 3-bp sequence upstream of the − 10 box was highly efficient to generate apparently stronger promoters.

**Fig. 3:**
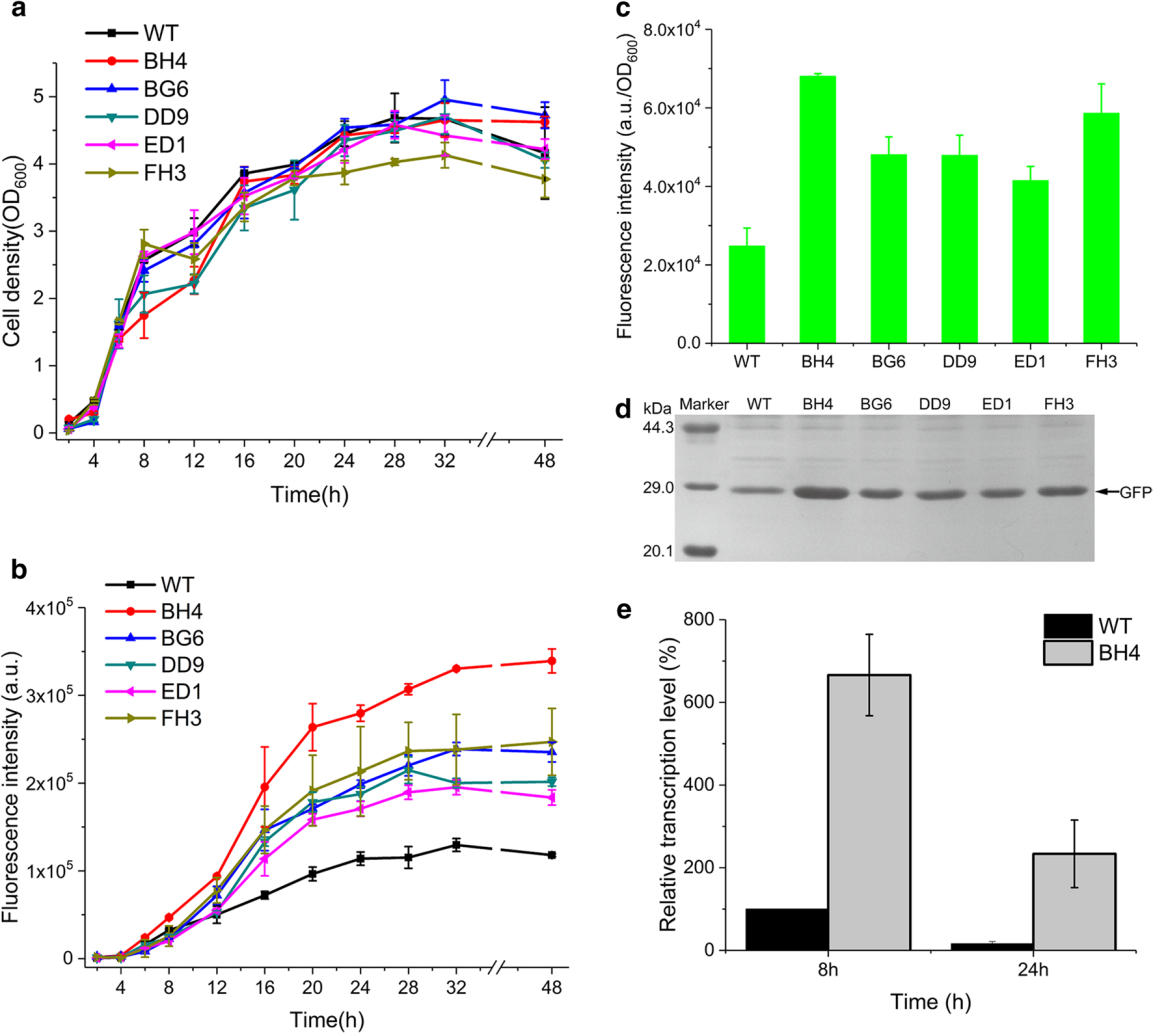
Characterization of the evolved mutants. **a** The growth curves of the five mutants. WT stands for the strain harboring the parental promoter. **b** The time-course GFP expression levels of these mutants were determined using the FI. **c** The expression levels of GFP of the five mutants were compared to the WT to reveal the superior mutant for further characterization and application. The FI values of the five mutants after the 28-h culture in the shake flask condition were adopted to compare the differences in GFP expression. The data are shown as the mean ± S.D. All the experiments were performed independently in triplicate. **d** SDS-PAGE analysis confirmed the expression levels of GFP of the five mutants. The whole-cell proteins of each sample were separated by 12% SDS-PAGE and were then stained with Coomassie Brilliant Blue R-250. **e** RT-qPCR was carried out to analyze the relative expression level of GFP under the control of WT and the variant promoter P_BH4_ at 8 h- and 24 h-culture. The data are calculated by the 2^−ΔΔCt^ method. All the data were independently repeated in triplicate

Furthermore, according to the strength determined in the 96-well plate, we selected 14 mutants, which covered higher and equivalent strength compared to the WT, to verify the expression levels. The FI value of each mutant showed that C12, H2, and H8 exhibited approximately equivalent strengths compared to the parental promoter, while B2, B8, B6, C1, D7, D8, D11, E9, F2, F4, G11, and H2 displayed a higher strength than the parental promoter to different degrees. Among of them, the promoter strength of four mutants in the pPML, D11, F2, E9, and G11, increased by more than 2 times compared to the parental promoter. Noticeably, E9 possessed the highest strength in the 96-well plate (Fig. 3c). The sequences for the 14 mutants were also shown to validate the mutation site (Additional file 1: Figure S3B).

To avoid the variation in strength in flask system, four typical mutants, exhibiting very high (P_E9_) strength, high (P_F2_) strength, medium high (P_D11_) strength, and slight high (P_B2_) strength, respectively, were chosen to further authenticate the mutant strength in shake flask. Noticeably, the relative GFP expression level driven by P_E9_, which had an FI of 57,386 ± 4504 after 24-h of culture, increased approximately by 2.2 times compared to that of P_srfA_. The FI values for P_B2_, P_D11_, and P_F2_ were 28,460 ± 617, 47,596 ± 36, 53,134 ± 552 and 57,386 ± 4504, respectively, after the same culture time (Fig. 2c). The tendency of the strength of the four mutants was consistent with that in the 96-well plate (Additional file 1: Figure S3C). SDS-PAGE analysis further confirmed the consistent results (Fig. 2d).

Accordingly, to further strengthen the activity of promoter, we set out to construct secondary PML (sPML) based on the pPML. We designed a random mutation targeting the 4-bp position upstream of − 16 box based on the mutants gained from the first-round evolution (Fig. 1b). We constructed four sPMLs, sPML1, sPML2, sPML3, and sPML4, employing the four firstly evolved mutants, P_B2_, P_D11_, P_F2_, and P_E9_, respectively. In this round of screening, we picked 90 transformants from each library and determined the relative expression level of GFP. By alignment of the strength of the selected mutants in each sPMLs, four diverse strength profiles were observed in this round of evolution. Finally, a total of 9 mutants exhibiting higher strength than the corresponding templates were obtained, among which 4 mutants were from sPML1 and others were from sPML2. However, none of further evolved mutants possessed higher strength than the template was screened in this round of evolution from sPML3 and sPML4 (Additional file 1: Figure S4A–D). Nevertheless, several mutants obtained from sPML3 and sPML4 displayed higher strength than that of the original parental promoter.

Subsequently, we selected a total of 12 mutants from the four sMPLs, including five and four mutants exhibiting the highest strength from sPML1 and sPML2, respectively. As well, one mutant and two mutants from sPML3 and sPML4, respectively, which also represented the highest strength, were also included. The sequences at the 4-bp position of these mutants were sequenced and aligned. These mutated sequences displayed high diversity compared to their original parental promoter, since the mutations accumulated by the second-round of evolution (Additional file 1: Figure S4E). These results indicate that the iterative mutagenesis, targeting the 4-bp position based on the templates of D11 and B2, further evolved the strength by the second round of evolution. The evolved performance of the promoter mutant accumulated due to the multiple mutagenesis that targeted the spacer sequence in the stepwise manner. However, the dynamic range profiles of sPML3 and sPML4 also revealed that the iterative mutagenesis targeting the position far away from the − 10 box had a limited evolutionary capacity, especially evolving the promoter that had an excellent performance.

These results from the two rounds of evolution suggest that the bacterial promoter is capable to be successfully evolved through the construction of a small promoter mutagenesis library targeting a few nucleotides upstream of the − 10 box by stepwise mutation. The typically utilized approach of randomizing the entire spacer to generate rather large variant libraries (SPL) produces a huge number of mutants in the library and requires multiple-round screening by fluorescence-activated cell sorting (FACS) [51, 60, 61], which is more suitable to fabricate a mutant pool displaying a dynamic range in strength, as large numbers of mutants screened from SPL will cover the strength from a rather low level to a high level. However, as to the purpose for obtaining higher-strength mutants, excessively screening of large-scale library absolutely generates a plethora of unnecessary clones, even none of evolved clone was gained. This limitation has been found in the recent reports. An extremely large library constructed by 70-bp random mutagenesis in *Corynebacterium glutamicum* was screened by FACS. However, only 20 promoters with activity were obtained [51]. Similarly, a large SPL based on promoter P43 was also constructed in *B. subtilis*. After screening of 5000 colonies, no mutants showing higher activity than that of WT were obtained [62]. In line with these results, our previous study found that mutagenesis targeting a total of 7-bp sequence upstream of the − 10 box of P_srfA_ scarcely obtained the strength-evolved mutants after screening of hundreds of clones. In contrast, SETarSCoP strategy would be a feasible and convenient strategy to evolve diverse bacterial promoters to expand the toolbox for synthetic biology.

### Characterization and verification of the evolved mutants from sPMLs

To better characterize the stability, robustness, and strength of these superior mutants after the two-round SETarSCoP screening, we chose five mutants with relatively higher strength than sPML1 and sPML2 to determine the performance in the batch condition along with the culture time. A consistent output, controlled by a strong promoter, is desired for the host harboring synthetic construct [47]. However, consistency is often confounded by the inherently stochastic nature of gene expression, which results from both promoters and any downstream cargo proteins that confer known effects to the host [63].Growth curves for the selected mutants were simultaneously profiled to estimate the expression burden to the host. The FI levels of P_BH4_ and P_BG6_ derived from P_B2_, P_DD9_ derived from P_D11_, P_ED1_ derived from P_E9_, and P_FH3_ derived from P_F2_, were measured and subsequently compared to the parental P_srfA_. The growth curves of the five mutants regularly increased with cultural time till the stationary phase without significant fluctuation might be rendered by the detrimental growth burden (Fig. 3a). The expression levels of GFP, driven by the five mutants, increased regularly along with the culture time, which showed a similar tendency but had a different overall level for each mutant. Compared to the expression profile of the parental P_srfA_, all five mutants evolved toward a higher strength without significantly altering the expression pattern (Fig. 3b). Among the selected mutants, P_BH4_ mediated the highest expression level of GFP after 28 h of culture, when the cells entered the steady stationary phase. The expression level was approximately 2.7 times higher than that of the parental P_srfA_ (Fig. 3c). SDS-PAGE further confirmed that the highest expression of GFP was by P_BH4_ (Fig. 3d). Furthermore, we verified that the functional evolution of P_BH4_ was performed at the transcriptional level. RT-qPCR data showed that the transcriptional activity of P_BH4_ was approximately higher than that of parental P_srfA_ by 7 and 15 times at the 8-h and 24-h culture, respectively (Fig. 3e), confirming that the evolution ascribed to the transcription driven by the mutagenesis occurring in the mutants, manifesting that the evolved mutants from the different sPMLs had better performance than the parental promoter. These data elucidate that the SETarSCoP strategy established in this study is a feasible and a simpler approach for the evolution of the synthetic bacterial promoters.

### Evaluation of the suitability of P_BH4_ to diverse cargo proteins

**Fig. 4:**
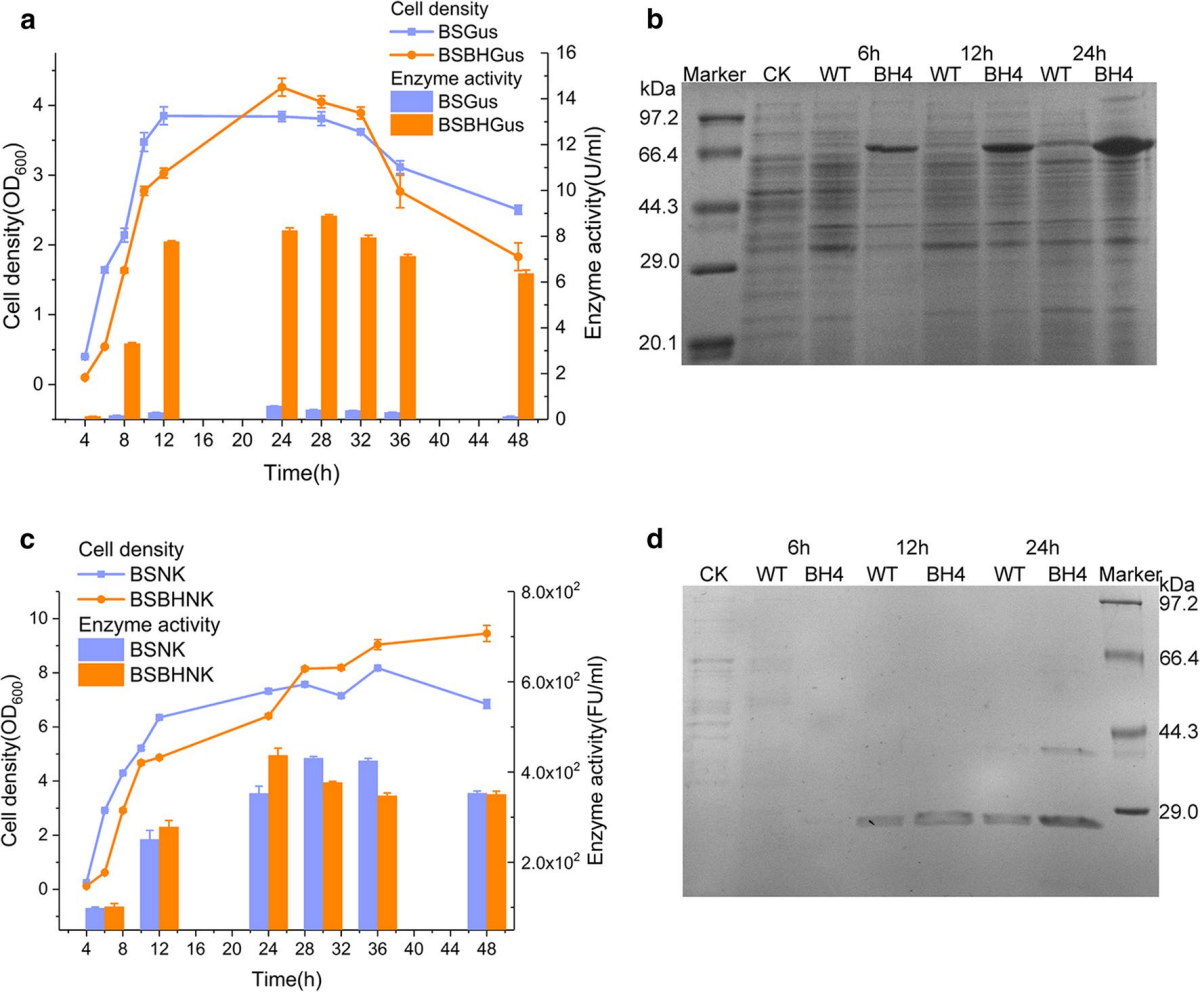
Heterologous protein expression in the shake flask condition identified the compatibility of the evolved promoter. **a** The growth curves and GusA expression levels of BSGus and BSBHGus at different culture times were measured and determined to show the promoter strength and the compatibility to the Gus protein. The data are shown as the mean ± S.D. All the experiments were performed in triplicate. **b** SDS-PAGE analysis confirmed the expression level of GusA. The treatment of the samples was performed as aforementioned. CK denoted the strain without any plasmid. **c** The growth curves and expression levels of NK of BSNK and BSBHNK, at the different culture times, were measured to identify the compatibility and stability of BH4 with the host. The data are shown as the mean ± S.D. All the experiments were performed in triplicate. **d** The expression levels of NK were determined by SDS-PAGE analysis after 6-, 12-, and 24-h culture. The treatment of each sample was performed as aforementioned. CK denoted the strain without any plasmid

According to the two-round directed evolution via SETarSCoP, the mutant promoter P_BH4_ was validated to possess the highest strength. In principle, the performance of a promoter, including its availability and robustness, is characterized and appraised by the over-expression of diverse proteins, usually employing disparate reporter proteins, industrial enzymes for biotransformation and for metabolic engineering, and pharmaceutical proteins [51, 53, 61, 62, 64].To verify the suitability of P_BH4_ to diverse target proteins, GusA was first employed to test whether it could be over-expressed at a level consistent with GFP. The expression level of GusA, under the control of P_srfA_ and P_BH4_, was measured at different time spans of the entire growth phase, with a total culture time of 48 h. The expression level of GusA in BSBHGus, harboring P_BH4_, maintained at a relatively higher level than that of P_srfA_, although, they had similar cell-growth curves over the culture time (Fig. 4a). The highest enzymatic activity of GusA produced by BSBHGus and BSGus was 8.9 ± 0.1 U/mL at 28 h and 0.6 U/mL at 24 h, respectively (Fig. 4a). SDS-PAGE analysis of the expression levels of GusA at the mid-exponential phase (6 h), late-exponential phase (12 h) and stationary phase (24 h) in the batch condition confirmed that P_BH4_ tremendously augmented the expression level of GusA (Fig. 4b).

To further evaluate whether P_BH4_ was suitable to the functional heterologous protein, NK from *Bacillus natto* was cloned and over-expressed in *B. subtilis* 3NA. Recombinant strains BSNK and BSBHNK harboring P_srfA_ and P_BH4_, respectively, had similar cell growth curves that regularly increased at the exponential phase. The divergent cell growth occurred at the stationary phase, during which the cell growth of BSNK slightly fluctuated, while the BSBHNK consistently increased over the whole culture period (Fig. 4c). The enzyme activity of NK, produced by BSBHNK, was higher than that of BSNK at 24 h, while it appeared to be lower at both 30 h and 36 h. The highest enzyme activities of BSNK and BSBHNK were 430.8 ± 3.5 FU/mL and 437.2 ± 15.8 FU/mL, respectively, which were equivalent to each other (Fig. 4c). This shorter time for reaching the highest enzyme activity indicated that the expression strength of NK in BSBHNK was greater than that in BSNK. These results indicate that P_BH4_ potentially has a broad scope of cargo proteins. SDS-PAGE analysis of the secretion of NK at 4, 12 and 24 h in the batch condition confirmed that P_BH4_ facilitated the production of NK compared to the parental promoter (Fig. 4d). The P_BH4_ synthetic promoter obtained in this study by SETarSCoP derived from the P_srfA_ is stronger than the previously reported mutants derived from the same parental template by only using a semi-rational design strategy targeting the conserved and non-conservative sequences, upon which the highest yielding level of NK controlled by that evolved promoter was 292 FU/mL at the similar culture condition [65]. Therefore, SETarSCoP is superior to several previous strategies for promoter evolution on different aspects.

### Proof-of-principle test of SETarSCoP by using two types of promoters

**Fig. 5:**
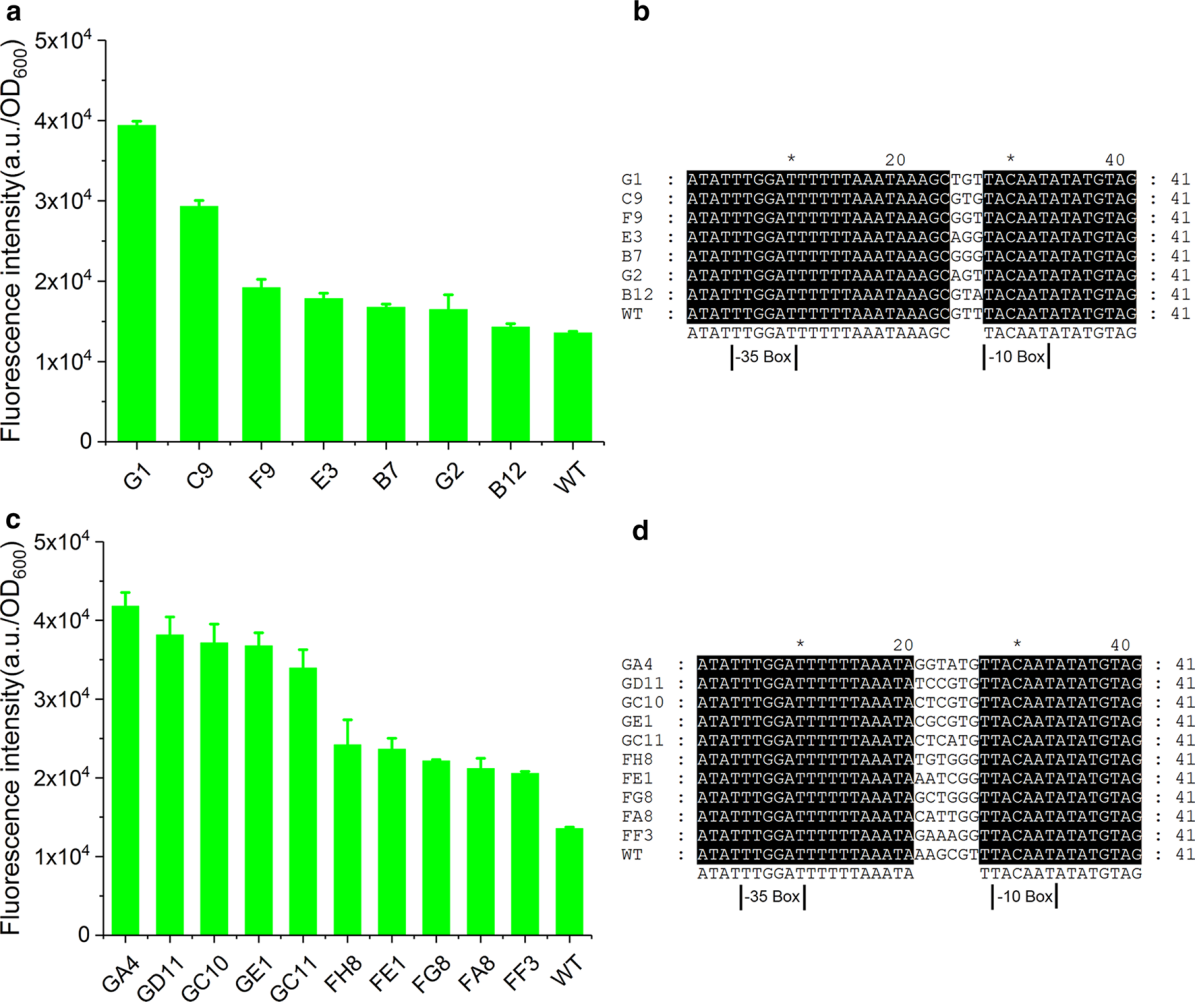
Proof-of-principle of SETarSCoP. **a** Characterization of the FI between the P_ylbP_ and the 7 mutants from the first round of evolution. **b** Sequence alignment of the mutants from the first round of evolution. **c** Characterization of the FI between the P_ylbP_ and 10 mutants from the second round of evolution. **d** Sequence alignment of the mutants from the second round of evolution

To verify the generalizability of SETarSCoP in evolution of bacterial promoter, we here applied this method to other two types of commonly used promoters, a constitutive promoter P_ylbP_ and a xylose-inducible promoter P_xylA_. After the first-round mutagenesis targeting the 3-bp adjacent to the − 10 box using SETarSCoP, a series of mutants displaying continuous strength were obtained, including 26 mutants with higher strength than the P_ylbP_ in the pPML (Additional file 1: Figure S5A). Among these mutants, G1 was highest in strength, which increased by 2 times compared to the P_ylbP_ (Fig. 5a). Sequencing of 7 mutants with higher strength in different degrees revealed that diversified nucleotide sequence occured at this position (Fig. 5b). Then, the second-round evolution on G1 (with the highest strength) and F9 (with medium strength) was performed, which targets the 4-bp immediately upstream of the first-round mutagenesis sites. We obtained several mutants with further enhanced strength from sPMLs (Additional file 1: Figure S5B and C). Five typical mutants selected from each sPML were compared with the wild-type promoter. Over-production of GFP by each of them displayed higher strength than the wild-type (Fig. 5c). Especially, the GA4 was higher than wild-type promoter by approximately 3 times. Sequencing results confirmed the diversity of the nucleotides in this region (Fig. 5d). In parallel, the P_xylA_ was also able to acquire higher strength through SETarSCoP. Mutants with significantly enhanced strength can be also obtained from pPML or sPMLs (Additional file 1: Figure S6). These results implied that diverse kinds of bacterial promoters, including the constitutive promoters and the inducible promoters can be effectively evolved via SETarSCoP.

## Methods

### Bacterial strains, plasmids and growth conditions

All the strains and plasmids used in this study are listed in Table 1. The plasmid pBSG03, a shuttle plasmid harboring the reporter gene *gfp* driven by promoter P_srfA_, was used as the template for constructing the mutant library of P_srfA_. A highly efficient competent *B. subtilis* comK, constructed by integrating the P_xylA_-*comK* operon into the *lac*A site, was used to screen mutant P_srfA_ with various activities (Additional file 1: Figure S1). The *spo*0*A* mutant type strain *B. subtilis* 3NA [66] was used to over-expression of NK. *B. subtilis* 3NA was purchased from Bacillus Genetic Stock Center (BGSC). *E. coli* JM109 was used as the host for the propagation of the recombinant plasmids and the construction of the mutant library. Luria–Bertani (LB) medium (10 g/L tryptone, 5 g/L yeast extract, 10 g/L NaCl, pH 7.0) was used to culture the hosts. Terrific-Broth (TB) medium (12 g/L tryptone, 24 g/L yeast extract, 4 g/L NaCl, 17 mM KH_2_PO_4_, and 72 mM K_2_HPO_4_) was used to culture the recombinant *B. subtilis* harboring the plasmid for the over-production of NK. The concentrations of the antibiotics for the selection and growth were added to the media as follows: 100 μg/mL ampicillin; 5 μg/mL kanamycin; and 1 μg/mL erythromycin.

**Table 1:** Plasmids and strains

Plasmids and strains	Relevant characteristics	References or source
Plasmids		
pAX01	*B. subtilis* integration vector, xyl^r^-P_xyl_ cassette, Amp^r^, Erm^r^	Lab stock
pAX-comK	Derived from pAX01, comK inserted	This study
pBP43GFP	*E. coli*-*B. subtilis* shuttle vector, P43 promoter, *gfp*, Amp^r^, Kan^r^	Lab stock
pBSG03	*E. coli*-*B. subtilis* shuttle vector, P_srfA_ promoter, *gfp*, Amp^r^, Kan^r^	Lab stock
pBS-gusA	*gusA* expressed by wild type P_srfA_	This study
pBBH4-gusA	*gusA* expressed by mutant promoter P_BH4_	This study
pBS-NK	Secretion expression of nattokinase by wild type P_srfA_ and WapA signal peptide	This study
pBBH4-NK	Secretion expression of nattokinase by wild type P_BH4_ and WapA signal peptide	This study
pBP_ylbP_-GFP	Derived from pBSG03, P_srfA_::P_ylbP_-RBS, *gfp*, Amp^r^, Kan^r^	This study
pBP_xylA_-GFP	Derived from pBSG03, P_srfA_::P_xylA_, *gfp*, Amp^r^, Kan^r^	This study
Strains		
*E.coli* JM109	*recA*1, *supE*44 *endA*1 *hsdR*17 (^r−^k,m^+^ k) *gyrA*96 *relA*1 thi (*lac*-*proAB*) F’[*traD*36 *proAB*^+^ *lacI*^q^ *lacZ* ΔM15]	Lab stock
*B. subtilis* 168	*trpC*2	Lab stock
*B. subtilis* comK	Derived from *B. subtilis* 168, *lacA*::P_xylA_-*comK*, *Erm*^r^	This study
*B. subtilis* 3NA	*spo*0A3	Lab stock
BSGus	*B. subtilis* comK harboring pBS-gusA	This study
BSBHGus	*B. subtilis* comK harboring pBBH4-gusA	This study
BSNK	*B. subtilis* 3NA harboring pBS-NK	This study
BSBHNK	*B. subtilis* 3NA harboring pBBH4-NK	This study

### Genetic manipulation

In this study, we constructed plasmids using the digestion-ligation and Gibson Assembly method according to a previous method with some modifications [67]. When using the Gibson Assembly, the recipient plasmid and the inserts were first amplified individually using PrimSTAR HS DNA Polymerase (Takara, Japan) according the product instructions. The PCR products were treated with *Dpn*I to eliminate the template prior to the purification, using the MagExtractor-PCR &Gel Clean up kit (TOYOBO, Japan). Finally, these two linearized fragments, with homologous arms, were seamlessly ligated and cycled with each other using the 1.33 × Master Mix.

### Construction of expression plasmids

The plasmids pBS-GusA and pBBH4-GusA (Table 1) were constructed to verify the compatibility of the constructed promoter variants P_BH4_ to the different cargo proteins. We amplified gene *gusA* from the *E. coli* JM109 chromosome using the primers P_gusA_-i1/P_gusA_-i2 (Additional file 1: Table S1), while the backbone of the plasmids, harboring P_srfA_ and P_BH4_, was amplified using the universal primers P_gusA_-v1 and P_gusA_-v2 (Additional file 1: Table S1). Then, *gusA* was inserted into the shuttle vectors using the Gibson assembly, yielding pBS-GusA and pBBH4-GusA. Accordingly, these two plasmids were transformed into *B. subtilis* (comK), yielding the recombinant hosts BSGus and BSBHGus.

The plasmid pBBH4-NK, harboring NK, was constructed to determine the expression level under the control of P_BH4_ in recombinant *B. subtilis*. The CDS of NK and the plasmid backbone harboring P_BH4_ were amplified from the plasmid pBS-NK, with the primer pair P_nk_-i1/P_nk_-i2 and P_nk_-v1/P_nk_-v2 (Additional file 1: Table S1), respectively. Then, the two PCR products were assembled by the Gibson Assembly as aforementioned, yielding pBBH4-NK. The construct was transformed into *B. subtilis* 3NA, and the resultant strains were designated BSNK and BSBHNK.

Plasmid pBP_xylA_-GFP was generated by assembly of P_xylA_ sequence cloned from pAX01 and the backbone of pBSG03 devoid of P_srfA_ together by Gibson Assembly using primers P_xylA_-i1/P_xylA_-i2 and P_xylA_-v1/P_xylA_-v2. Plasmid pBP_ylbP_-GFP was constructed by a whole plasmid inverse PCR as previously reported [68], in which the minimal core region of P_ylbP_ including − 35 box, − 10 box and transcription start site was synthesized and then cloned into the pBSG03 lacking of P_srfA_.

### Construction and screening of the promoter mutagenesis libraries based on the SETarSCoP strategy

We introduced randomized mutations into the targeted region of P_srfA_ with degenerate oligonucleotides N using the plasmid pBSG03 as the template. The PCR amplification protocol was performed according to the guidance provided by the QuikChange™ site-directed mutagenesis method [69]. The PCR reaction was carried out for 18 cycles as follows: heat denaturation at 98 °C for 15 s; annealing at 50 °C for 30 s and extension at 72 °C for 7 min. The PCR products were treated with *Dpn* I to eliminate the templates and were purified before they were transformed into *E. coli* JM109. Finally, the transformed cells were spread onto LB agar plates with ampicillin.

All the *E. coli* transformants were washed with sterile ddH_2_O followed by the extraction of the total variant plasmids. Then, we transformed the plasmids into *B. subtilis* comK and spread the transformed cells onto LB agar plates with kanamycin. Single clones were picked randomly and were placed into 96-deep-well plates containing 600 μL LB media with kanamycin and were cultured at 37 °C and 800 rpm. After 24 h of growth, 200 μL of the bacteria liquid was transferred into a black-wall 96-well plate, and then, the fluorescence intensity (FI) of GFP and the OD_600_ was measured with the Synergy ™ H4 multimode microplate reader (BioTek Instruments, Inc., USA). For screening of promoter mutagenesis of P_ylbP_ and P_xylA_, plasmid pBP_ylbP_-GFP and PBP_xylA_-GFP were used as templates, and the workflow was same with that of P_srfA_.

### Transformation of competent *B. subtilis* comK cells

Single clones were cultured overnight at 37 °C with rigorous shaking in the tube containing 5 mL LB medium. The overnight culture was diluted at a ratio of 1:50 prior to being transferred into fresh LB, and then, it was cultured at 37 °Cwith rigorous shaking. The cell density of the culture was monitored over time until the OD_600_ reached 0.4, after which xylose, at a final concentration of 1%, was added and treated for 2 h to induce the cells to enter the competence state. The prepared competent cells were divided into small aliquots and stored at − 80 °C. For the transformation, 3 μL of and one aliquot of cells were mixed together and incubated at 37 °C for 3 h with 200 rpm shaking. The transformed cells were then spread onto LB agar plates with the appropriate antibiotics.

### GFP expression and fluorescence assay

Single clones of the recombinant *B. subtilis* strains harboring the recombinant plasmids were inoculated into the test tubes containing 5 mL of LB medium and cultured overnight at 37 °C with rigorous shaking at 200 rpm. Then, cells (the initial OD_600_ approximately was 0.05) were transferred into a 250-mL shake flask, containing 50 mL of fresh LB, after which the cultures were incubated at 37 °C with shaking at 200 rpm. The cultures were periodically sampled to monitor the growth and GFP expression. The cells were harvested by centrifugation at 8000 rpm for 5 min, and the pellet was washed three times prior to being resuspended in phosphate-buffered saline (PBS, 8 g/L NaCl, 0.2 g/L KCl, 1.44 g/L Na_2_HPO_4_, and 0.24 g/L KH_2_PO_4_, pH 7.4). Each sample (200 μL) was transferred into a 96-well black-walled plate and was analyzed by a Synergy ™ H4 multimode microplate reader (excitation 495 nm, emission 525 nm).

### Analysis of conserved sequence of promoters associated to transcription level

Time-Resolved Transcriptomics data were extracted from Gene Expression Omnibus (GEO) of National Center for Biotechnology Information Search database (NCBI) (GEO accession: GSE19831) [59]. The strength of those promoters was ranked by the transcriptional levels over culture process. The spacer sequences between − 35 box and − 10 box of these selected promoters were gained from DBTBS database (http://dbtbs.hgc.jp/), and the sequence conservation was analyzed by WebLogo (http://weblogo.berkeley.edu/logo.cgi).

### Transcriptional level analysis by RT-qPCR

We used RT-qPCR to evaluate the difference in the transcriptional level driven by the wild type promoter and the mutants. The 16S rDNA gene was chosen as the internal reference. The total RNA was extracted from the cells using the RNAprep Pure Cell/Bacteria Kit (TIANGEN BIOTECH Co., Ltd., Beijing, China). The Reverse transcription was performed with the PrimeScript ™ RT reagent Kit with gDNA Eraser (Perfect Real Time) (Takara, Japan). Then, the real time PCR was performed with the SYBR^®^Premix Ex Taq ™ II (Tli RNaseH Plus) (Takara, Dalian, China) and the CFX96 Touch™ Real-Time PCR Detection System (Bio-Rad Laboratories, Inc., USA). The real-time PCR program was as follows: 95 °C for 30 s; then 40 cycles of 95 °C for 5 s and 60 °C for 30 s; then 95 °C for 10 s; then 60 °C for 30 s and increasing to 95 °C to test the melt curve. The primers P_qGFP_-1 and P_qGFP_-2 were used for the gfp gene test, and the primers P_q16S_-1 and P_q16S_-2 were for the 16S rDNA gene (Additional file 1: Table S1). Finally, the data are represented by the 2 ^−ΔΔCt^ method.

### Enzymatic activity assay of GusA and NK

To verify the expression level of GusA, driven by P_srfA_ and P_BH4_, BSGus and BSBHGus were cultured in 250-mL conical flasks with a working volume of 50 mL of LB. The activity of GusA was measured with 4-nitrophenyl β-d-glucuronide (PNPG) as described previously [7]. Strains BSNK and BSBHNK were used to verify the expression level of NK, driven by P_srfA_ and P_BH4_ and NK activity was determined by a modified fibrin degradation assay [65].

## Additional file

**Additional file 1: Figure S1.** Transcriptional level analysis of strong and weak promoters. The transcriptional level of the 50 strongest promoters (A) and the 50 weakest promoters (B) were analyzed and shown by heatmap. Data were obtained from Gene Expression Omnibus (GEO) of National Center for Biotechnology Information Search database (NCBI) (GEO accession: GSE19831). Names of genes were labeled on the left of figures, and the serial numbers above means various growth time of samples. **Figure S2.** Construction and optimization of high-efficient transformation system in *Bacillus subtilis*. The competence transcription factor ComK was under control of xylose operon, which was integrated into the lacA site through double cross (A). Three factors: OD_600_ when added the xylose (B), concentration of xylose (C) and induction time (D), that influenceing transformation efficiency were optimized. Error bars are the s.d. of three independent experiments. **Figure S3.** Screening of pPML of P_srfA_. (A) One hundred and eighty-five transformants were screened from the MPL. The fluorescence intensity (FI) of all the mutants was ranked from the highest level to the lowest level. Fourteen mutants were denoted on the corresponding columns, which were selected for furthure verification and characterization. (B) Sequence alignment of the mutants from the first round of evolution. (C) Characterization of the FI of the parental promoter and 14 mutants from the first round of evolution. The dotted line stands for the 2-fold threshold of the FI compared to the parental promoter. **Figure S4.** Screening of sPML of P_srfA_. Fabrication and characterization of the four sMPLs, including MPL1, MPL2, MPL3, and MPL4, based on the random mutagenesis of the adjacent four nucleotides immediately upstream of the positions in the first-round evolution. The transformants obtained from the four MPLs based on the second round of evolution were screened in 96-well plates, and the gradient FI of these mutants in each MPL was ranked from the highest level to the lowest. The results were arranged by their templates, B2 (A), D11(B), F2 (C) and E9 (D), which were obtained from the first round of evolution. (E) Sequence alignment of the selected mutants from the MPL1 to MPL4 based on the second-round evolution. (F) Determination of the FI of the 12 mutants from the four sMPLs based on the second-round evolution. The dotted line stands for the 3-fold FI compared to the WT. **Figure S5**. Preliminary screening of sPML and pPML of P_ylbP_. (A) Screening of one hundred and eighty-five mutants of PylbP from MPL generated by the first round of mutagenesis targeting the 3 bp sequence adjacent to the upstream of -10 box. The fluorescent intensity (FI) of all the mutants was ranked from the highest level to the lowest level. (B) and (C) Fabrication and screening of the two sMPLs generated by random mutagenesis targeting the 4 bp adjacent to the upstream of first-round mutated region of PylbP-G1 and PylbP-F9 by SETarSCoP. **Figure S6.** Engineering of P_xylA_ through SETarSCoP. (A) One hundred and eighty-five transformants were screened from the MPL of P_xylA_. The FI of all the mutants was ranked from the highest level to the lowest level. (B) Characterization of the FI of the parental promoter and 6 mutants from the first round of evolution. (C) Sequence alignment of the mutants from the first round of evolution. (D) and (E) Fabrication and screening of the two sMPLs from P_xylA_–D7 and P_xylA_–D5. **Table S1**. Primers.

## Data Availability

All data generated or analysed during this study are included in this published article.
