# Supplementary material for: Development of a novel strategy for robust synthetic bacterial promoters based on a stepwise evolution targeting the spacer region of the core promoter in Bacillus subtilis
Source: Microb Cell Fact. 2019 May 29;18:96. doi: 10.1186/s12934-019-1148-3 (PMC6540529; doi:10.1186/s12934-019-1148-3)
Supplement: Supplementary file 1 — Additional file 1: Figure S1. Transcriptional level analysis of strong and weak promoters. The transcriptional level of the 50 strongest promoters (A) and the 50 weakest promoters (B) were analyzed and shown by heatmap. Data were obtained from Gene Expression Omnibus (GEO) of National Center for Biotechnology Information Search database (NCBI) (GEO accession: GSE19831). Names of genes were labeled on the left of figures, and the serial numbers above means various growth time of samples. Figure S2. Construction and optimization of high-efficient transformation system in Bacillus subtilis. The competence transcription factor ComK was under control of xylose operon, which was integrated into the lacA site through double cross (A). Three factors: OD600 when added the xylose (B), concentration of xylose (C) and induction time (D), that influenceing transformation efficiency were optimized. Error bars are the s.d. of three independent experiments. Figure S3. Screening of pPML of PsrfA. (A) One hundred and eighty-five transformants were screened from the MPL. The fluorescence intensity (FI) of all the mutants was ranked from the highest level to the lowest level. Fourteen mutants were denoted on the corresponding columns, which were selected for furthure verification and characterization. (B) Sequence alignment of the mutants from the first round of evolution. (C) Characterization of the FI of the parental promoter and 14 mutants from the first round of evolution. The dotted line stands for the 2-fold threshold of the FI compared to the parental promoter. Figure S4. Screening of sPML of PsrfA. Fabrication and characterization of the four sMPLs, including MPL1, MPL2, MPL3, and MPL4, based on the random mutagenesis of the adjacent four nucleotides immediately upstream of the positions in the first-round evolution. The transformants obtained from the four MPLs based on the second round of evolution were screened in 96-well plates, and the gradient FI of these mutants in each [file 12934_2019_1148_MOESM1_ESM.docx]

**Additional file**

**Development of a novel strategy for robust synthetic bacterial promoters based on a stepwise evolution targeting the spacer region of the core promoter in *Bacillus subtilis***

Laichuang Han, Wenjing Cui^*^, Feiya Suo, Shengnan Miao, Wenliang Hao, Qiaoqing Chen, Junling Guo, Zhongmei Liu, Li Zhou, Zhemin Zhou ^*^

School of Biotechnology, Key Laboratory of Industrial Biotechnology (Ministry of Education), Jiangnan University, Wuxi, Jiangsu, 214122, China

* To whom the correspondence should be addressed

Wenjing Cui: wjcui@jiangnan.edu.cn

Zhemin Zhou: zhmzhou@jiangnan.edu.cn

Tel: +86 (510) 8532 5210; Fax: +86 (510) 8519 7551

Laichuang Han: hanlaichuang@163.com

Feiya Suo: 1921508897@qq.com

Shengnan Miao: snanmiao1120@163.com

Wenliang Hao: 903070933@qq.com

Qiaoqing Chen: Chenqq18@163.com

Junling Guo:guojunling@jiangnan.edu.cn

Zhongmei Liu: zliu@jiangnan.edu.cn

Li Zhou: lizhou@jiangnan.edu.cn

**Supplementary Figures**


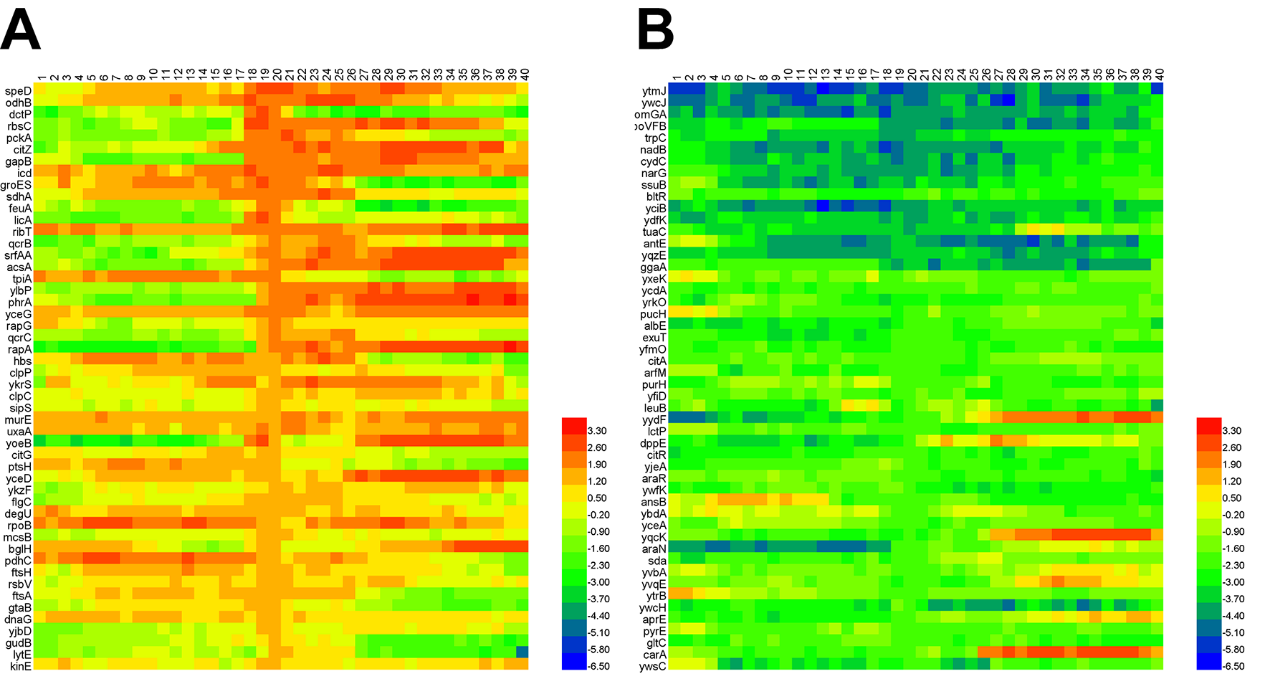


Figure S1 Transcriptional level analysis of strong and weak promoters. The transcriptional level of the 50 strongest promoters (A) and the 50 weakest promoters (B) were analyzed and shown by heatmap. Data were obtained from Gene Expression Omnibus (GEO) of National Center for Biotechnology Information Search database (NCBI) (GEO accession: GSE19831). Names of genes were labeled on the left of figures, and the serial numbers above means various growth time of samples.


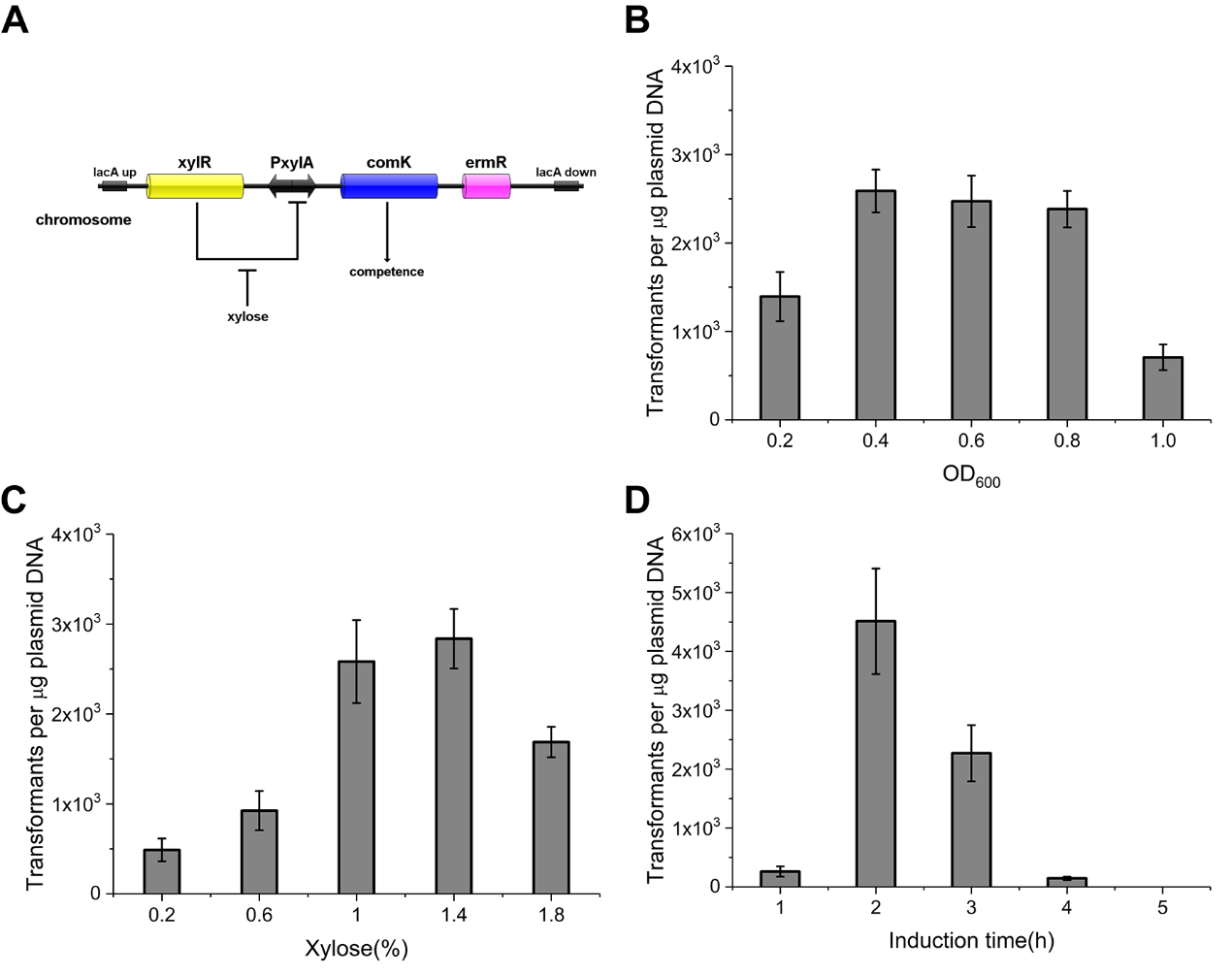


Figure S2 Construction and optimization of high-efficient transformation system in *Bacillus subtilis*. The competence transcription factor ComK was under control of xylose operon, which was integrated into the lacA site through double cross (A). Three factors: OD_600_ when added the xylose (B), concentration of xylose (C) and induction time (D), that influenceing transformation efficiency were optimized. Error bars are the s.d. of three independent experiments.


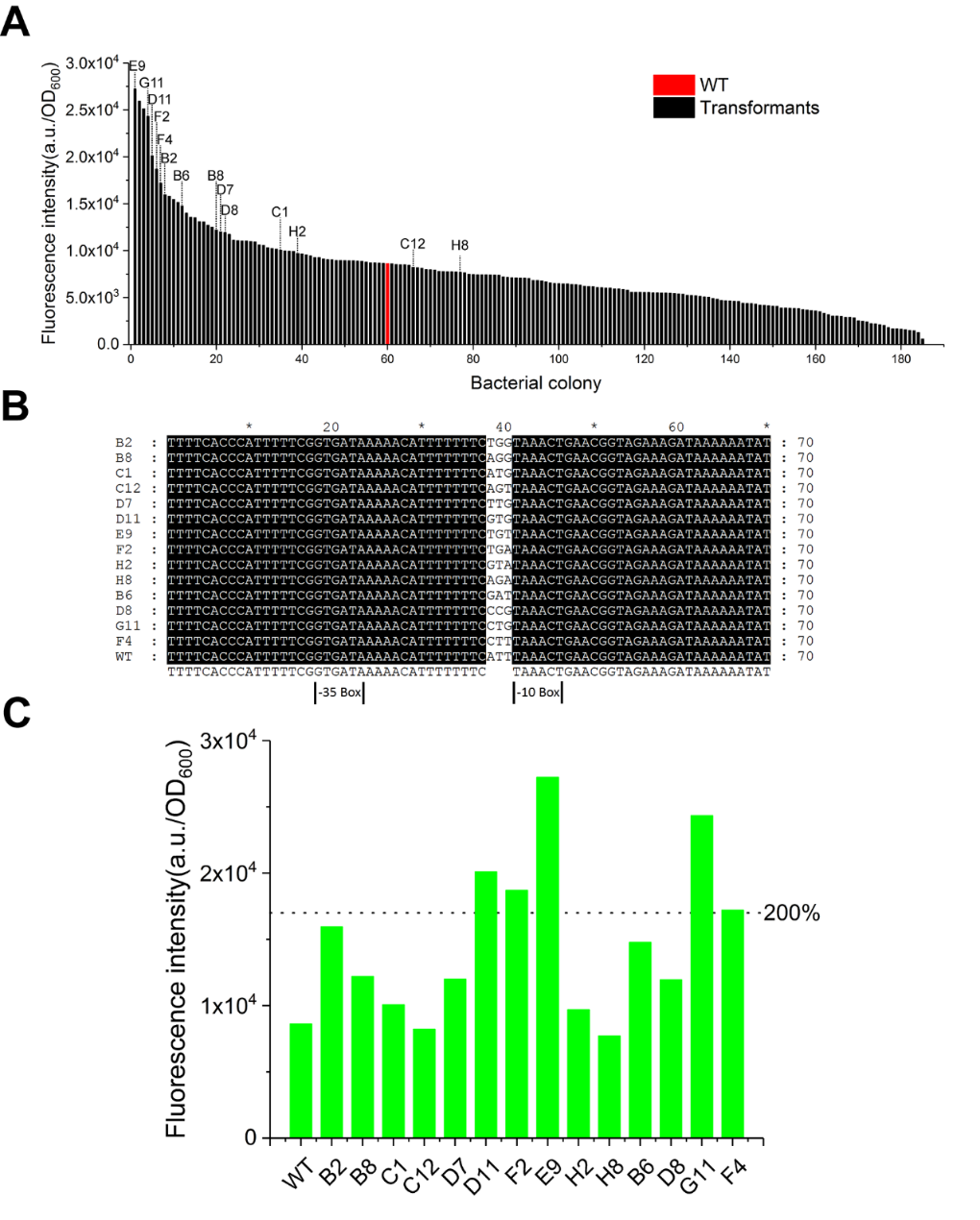


Figure S3 Screening of pPML of P_srfA_. (A) One hundred and eighty-five transformants were screened from the MPL. The fluorescence intensity (FI) of all the mutants was ranked from the highest level to the lowest level. Fourteen mutants were denoted on the corresponding columns, which were selected for furthure verification and characterization. (B) Sequence alignment of the mutants from the first round of evolution. (C) Characterization of the FI of the parental promoter and 14 mutants from the first round of evolution. The dotted line stands for the 2-fold threshold of the FI compared to the parental promoter.


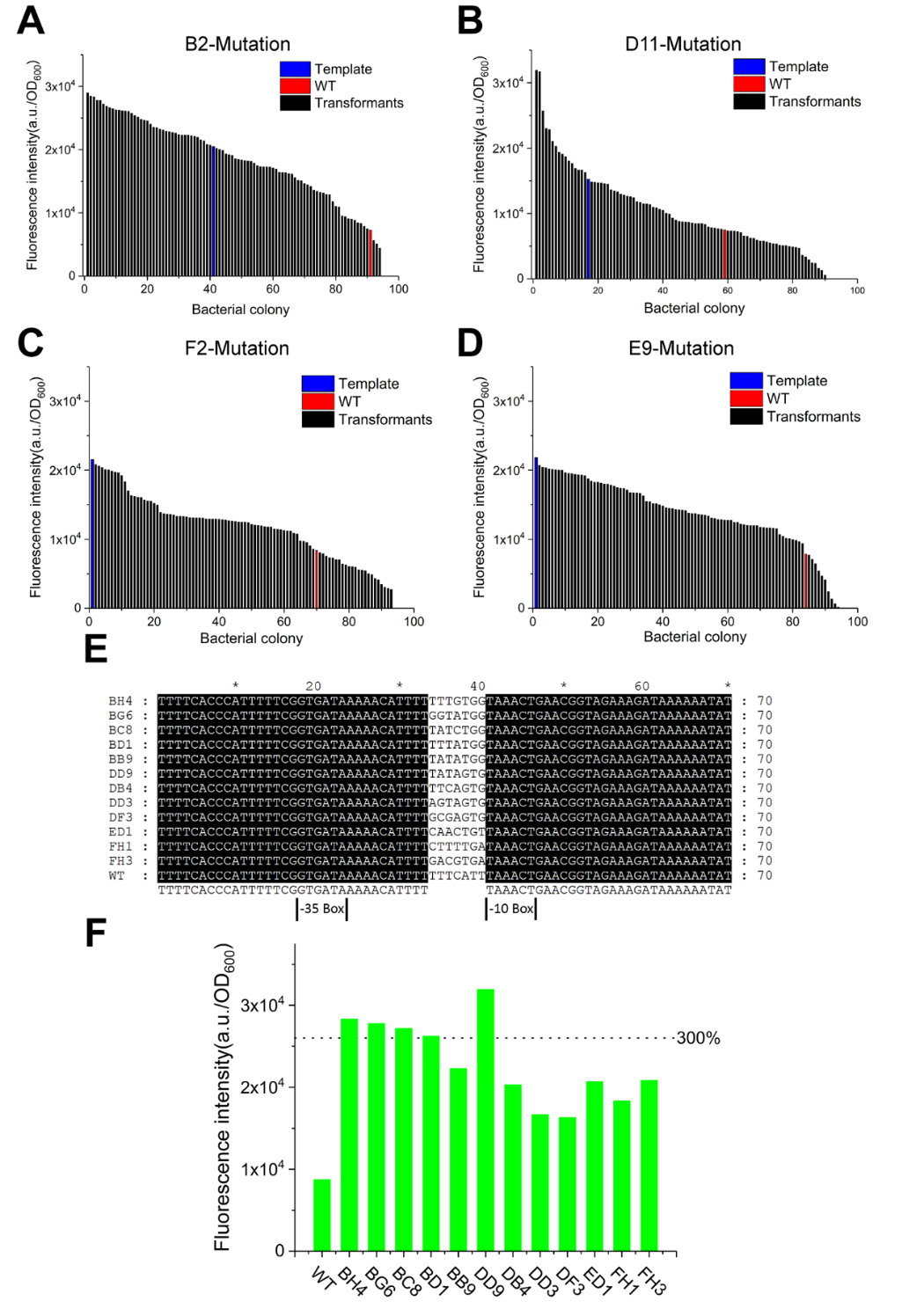


Figure S4 Screening of sPML of P_srfA_. Fabrication and characterization of the four sMPLs, including MPL1, MPL2, MPL3, and MPL4, based on the random mutagenesis of the adjacent four nucleotides immediately upstream of the positions in the first-round evolution. The transformants obtained from the four MPLs based on the second round of evolution were screened in 96-well plates, and the gradient FI of these mutants in each MPL was ranked from the highest level to the lowest. The results were arranged by their templates, B2 (A), D11(B), F2 (C) and E9 (D), which were obtained from the first round of evolution. (E) Sequence alignment of the selected mutants from the MPL1 to MPL4 based on the second-round evolution. (F) Determination of the FI of the 12 mutants from the four sMPLs based on the second-round evolution. The dotted line stands for the 3-fold FI compared to the WT.


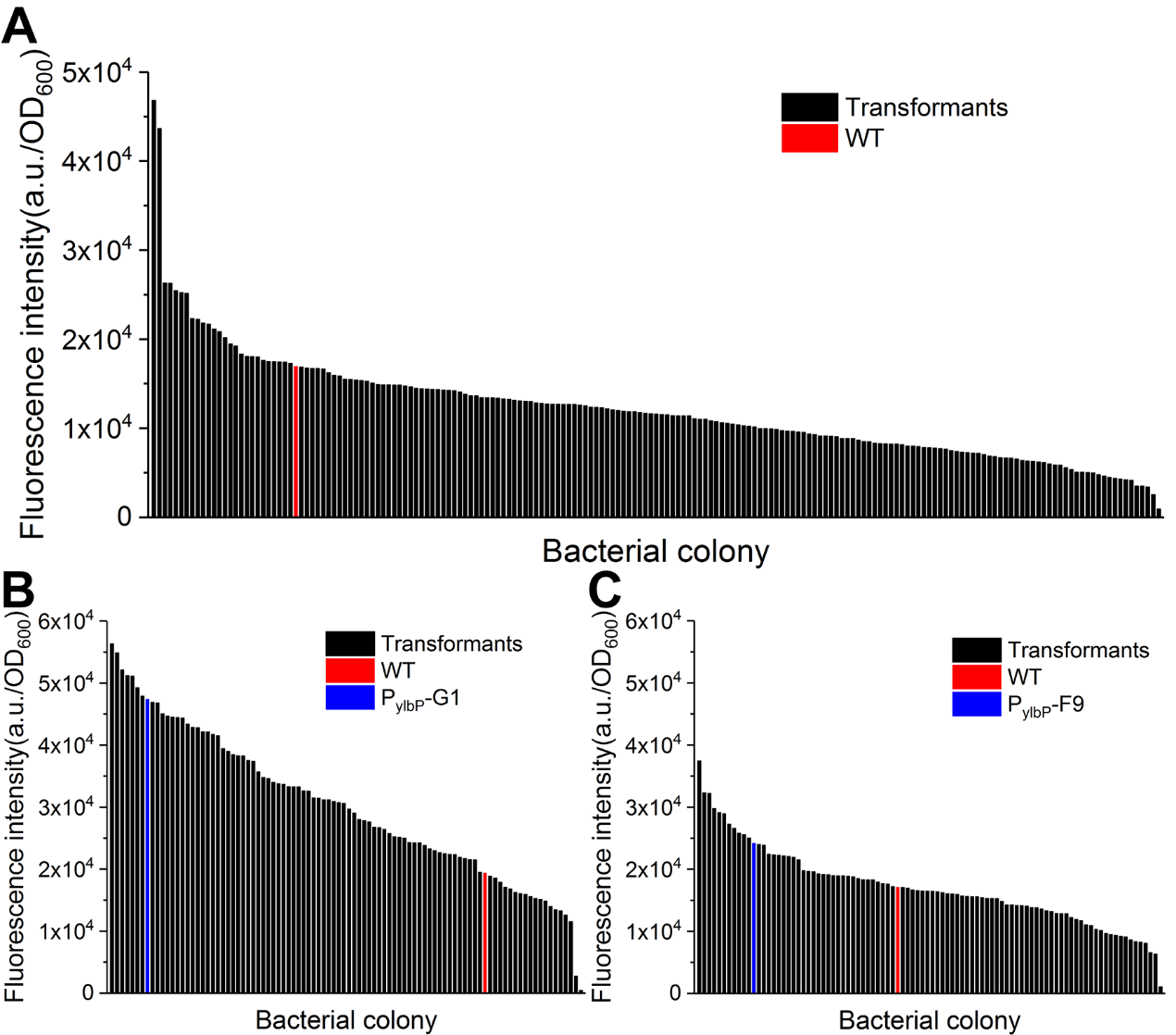


Figure S5 Preliminary screening of sPML and pPML of P_ylbP_. (A) Screening of one hundred and eighty-five mutants of PylbP from MPL generated by the first round of mutagenesis targeting the 3 bp sequence adjacent to the upstream of -10 box. The fluorescent intensity (FI) of all the mutants was ranked from the highest level to the lowest level. (B) and (C) Fabrication and screening of the two sMPLs generated by random mutagenesis targeting the 4 bp adjacent to the upstream of first-round mutated region of PylbP-G1 and PylbP-F9 by SETarSCoP.


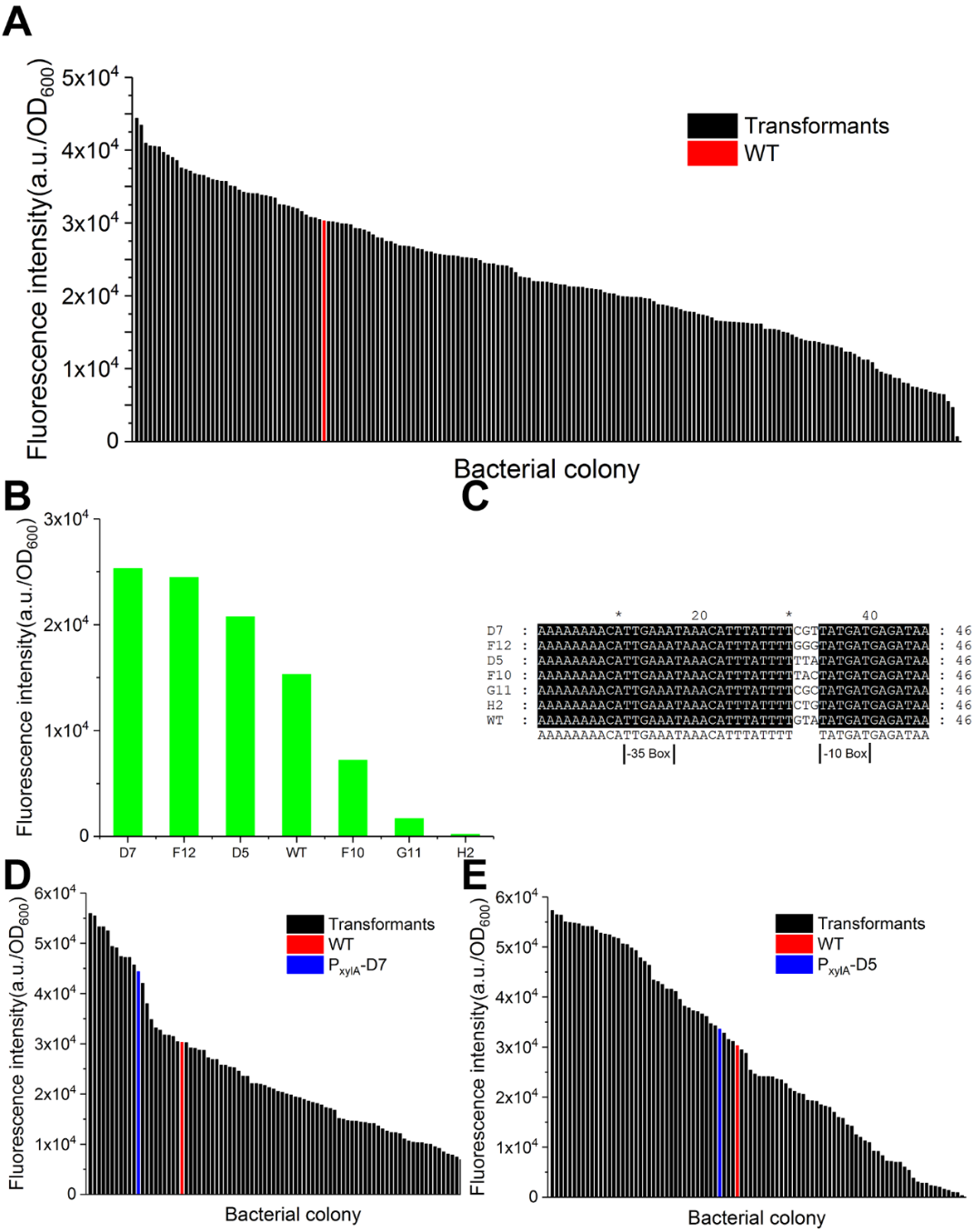


Figure S6 Engineering of P_xylA_ through SETarSCoP. (A) One hundred and eighty-five transformants were screened from the MPL of P_xylA_. The FI of all the mutants was ranked from the highest level to the lowest level. (B) Characterization of the FI of the parental promoter and 6 mutants from the first round of evolution. (C) Sequence alignment of the mutants from the first round of evolution. (D) and (E) Fabrication and screening of the two sMPLs from P_xylA_–D7 and P_xylA_–D5.

**Supplementary Table**

Table S1 Primers

| Primers | Sequences (5’-3’) |
| --- | --- |
| P_comK_-1  P_comK_-2  P_srfA_-3bp-1  P_srfA_-3bp-2  P_B2_-1  P_B2_-2  P_D11_-1  P_D11_-2  P_E9_-1  P_E9_-2  P_F2_-1  P_F2_-2  P_gusA_-i1  P_gusA_-i2  P_gusA_-v1  P_gusA_-v2  P_nk_-i1  P_nk_-i2  P_nk_-v1  P_nk_-v2  P_qGFP_-1  P_qGFP_-2  P_q16S_-1  P_q16S_-2  P_ylbP_-1  P_ylbP_-2  P_yblP_-3bp-1  P_yblP_-3bp-2  P_yblP_-G1-4bp-1  P_yblP_-G1-4bp-2  P_yblP_-F9-4bp-1  P_yblP_-F9-4bp-2  P_xylA_-i1  P_xylA_-i2  P_xylA_-v1  P_xylA_-v2  P_xylA_-3bp-1  P_xylA_-3bp-2  P_xylA_-D7-4bp-1  P_xylA_-D7-4bp-2  P_xylA_-D5-4bp-1  P_xylA_-D5-4bp-2 | CTAAAAATCAAAGGGGGAAATGGGATCCAAAGGAGGCCATAATATGAGTCAG  GGAAGAGTGCGGCCGCCCGCGGGAGCCTTCCTATTTTTCTAATACCGTTCC  GGTGATAAAAACATTTTTTTCNNNTAAACTGAACGGTAGAAAG  CTTTCTACCGTTCAGTTTANNNGAAAAAAATGTTTTTATCACC  CGGTGATAAAAACATTTTNNNNTGGTAAACTGAACGG  CCGTTCAGTTTACCANNNNAAAATGTTTTTATCACCG  CGGTGATAAAAACATTTTNNNNGTGTAAACTGAACGG  CCGTTCAGTTTACACNNNNAAAATGTTTTTATCACCG  CGGTGATAAAAACATTTTNNNNTGTTAAACTGAACGG  CCGTTCAGTTTAACANNNNAAAATGTTTTTATCACCG  CGGTGATAAAAACATTTTNNNNTGATAAACTGAACGG  CCGTTCAGTTTATACNNNNAAAATGTTTTTATCACCG  GATTAGGGGAGGTATGACAATGTTACGTCCTGTAGAAACCCCAACC  CCGCATCAGGCGAATTCCTACCGGCCGCATAGG  CCTATGCGGCCGGTAGGAATTCGCCTGATGCGG  GGTTGGGGTTTCTACAGGACGTAACATTGTCATACCTCCCCTAATC  TTAGGGGAGGTATGACAATGATGAAAAAAAG  ATACCGCATCAGGCGAATTC  TACAAGCAGCTGCACAATAAGAATTCGCCTGATGCGGT  CGCCTCTTTCTTTTTTTCATCATTGTCATACCTCCCCTAATCTTTATAAGC  AGATGGAAGCGTTCAACTA  GTGTGGACAGGTAATGGT  AAGTCCCGCAACGAGCGCAA  TCGCGGTTTCGCTGCCCTTT  TTTTTTAAATAAAGCGTTTACAATATATGTAGAAACAACAATCGAACATCATATTTAAAGTAAGAGGAG  ATATTGTAAACGCTTTATTTAAAAAATCCAAATATTTAAACTTTAACCTGCCCTCTGCCACC  TTTTTTAAATAAAGCNNNTACAATATATGTAGAAACAACAATCGAAC  TCTACATATATTGTANNNGCTTTATTTAAAAAATCCAAATATTTAAAC  TGGATTTTTTAAATANNNNTGTTACAATATATGTAGAAACAACAATCG  ACATATATTGTAACANNNNTATTTAAAAAATCCAAATATTTAAACTTTAAC  TGGATTTTTTAAATANNNNGGTTACAATATATGTAGAAACAACAATCG  ACATATATTGTAACCNNNNTATTTAAAAAATCCAAATATTTAAACTTTAAC  GAGGTGGCAGAGGGCAGGTCTAACTTATAGGGGTAACACTTAAAAAAGAATC  CAGTGAAAAGTTCTTCTCCTTTACTCATTTGTCATTTCCCCCTTTGATTTAAG  CTTAAATCAAAGGGGGAAATGACAAATGAGTAAAGGAGAAGAACTTTTCACTG  GATTCTTTTTTAAGTGTTACCCCTATAAGTTAGACCTGCCCTCTGCCACCTC  ATAAACATTTATTTTNNNTATGATGAGATAAAGTTAGTTTATTGG  CTTTATCTCATCATANNNAAAATAAATGTTTATTTCAATGTTTTTTTTAG  TGAAATAAACATTTANNNNCGTTATGATGAGATAAAGTTAGTTTATTGG  TATCTCATCATAACGNNNNTAAATGTTTATTTCAATGTTTTTTTTAGAAAATTTAG  TGAAATAAACATTTANNNNTTATATGATGAGATAAAGTTAGTTTATTGG  TATCTCATCATATAANNNNTAAATGTTTATTTCAATGTTTTTTTTAGAAAATTTAG |

Note: N stands for 4 base (A, T, C, G) with same probability.
